# Amphiphilic Copolymer of Polyhedral Oligomeric Silsesquioxane (POSS) Methacrylate for Solid Dispersion of Paclitaxel

**DOI:** 10.3390/ma12071058

**Published:** 2019-03-30

**Authors:** Suchismita Chatterjee, Tooru Ooya

**Affiliations:** Graduate School of Engineering, Department of Chemical Science and Engineering, Kobe University, Kobe 657-8501, Japan; suchismita@stu.kobe-u.ac.jp

**Keywords:** 2-methacryloyloxyethyl phosphorylcholine (MPC), polyhedral oligomeric silsesquioxane (POSS), polyvinylpyrrolidone (PVP), paclitaxel, solid dispersion, dissolution

## Abstract

Suitable polymers for the homogeneous formulation of drug/polymer mixtures should be selected to correct the structural and physicochemical nature with a rapid dissolution rate. This study aimed to evaluate a copolymer prepared by the radical polymerization of 2-methacryloyloxyethyl phosphorylcholine (MPC) and a polyhedral oligomeric silsesquioxane (POSS) methacrylate bearing an ethyl (C_2_H_5_) group (MPC-*ran*-C_2_H_5_-POSS) as a carrier for the solid formulation of paclitaxel (PTX). A single-phase homogeneous formulation of PTX with the mixture of the MPC-*ran*-C_2_H_5_-POSS and polyvinylpyrrolidone (PVP) was prepared by a solvent method. The formulation of MPC-*ran*-C_2_H_5_-POSS/PVP/PTX enhanced the dissolution rate and the dissolved amount (approximately 90% within 40 min) without precipitation. The X-ray diffraction (XRD), Fourier transform infrared spectroscopy (FT-IR) and differential scanning calorimetry (DSC) analysis confirmed the presence of PTX as an amorphous state. The amphiphilic nature of the MPC-*ran*-C_2_H_5_-POSS contributed to enhancing the aqueous solubility of PTX. The new formulation is applicable for solid dispersion technique via the supersaturation of PTX in an aqueous media.

## 1. Introduction

Paclitaxel (PTX) is well known as an excellent clinical agent against various types of cancer such as breast, ovarian, stomach and lung cancers, etc. Due to the hydrophobic nature and low aqueous solubility (~0.4 μg/mL), PTX is incorporated in a mixture of Cremophor EL (polyoxyethylated castor oil) and dehydrated ethanol for intravenous administration. However, the solvent is known to cause a serious hypersensitivity reaction [1]. In order to improve this serious problem, various formulations of PTX have been investigated using biocompatible materials and different methods of administration. Solid dispersion technology using biocompatible materials has been extensively studied to improve the solubility and dissolution of poorly soluble drugs, including PTX [2,3]. For example, PTX was incorporated into poly(ϵ-caprolactone)-based film [4]. A PTX-encapsulated liposome was investigated to improve its aqueous solubility, stability and clinical efficacy [5,6]. Nanoplatforms including nanoparticles (Abraxane^Ⓡ^) and micelles (Genexol^Ⓡ^, Nanoxel^Ⓡ^ and Paclical^Ⓡ^) have been used in clinical studies [7,8]. Alternatively, cyclodextrin complexes have been reported as formulations to increase water solubility [9,10], which has contributed to a decrease in toxicity. However, promising clinical effects have not yet been achieved.

Oral administration of PTX is important for the successful implementation of low-dose metronomic (LDM) chemotherapy, where relatively low doses of the drug are frequently administered with no drug-free periods, in terms of patient convenience and compliance. The major limitation of oral administration is the poor aqueous solubility of PTX, leading to poor bioavailability [11]. Recent studies suggest that a PTX-loaded glycyrrhizic acid (GA) micelle improved the oral bioavailability of PTX [12]. From the viewpoint of developing solid dispersion, suitable polymers to prepare the best solid dispersion should be designed depending on the chemical structure of poorly soluble drugs. For example, a copolymer composed of a 2-methacryloyloxyethyl phosphorylcholine (MPC) unit and a butyl methacrylate (BMA) unit (poly[MPC-*co*-BMA]) has been prepared [13], and the polymer spontaneously formed a micelle-like structure in an aqueous condition, where the hydrophobic domain could hydrophobically interact with PTX to solubilize it [14]. The poly[MPC-*co*-BMA] was also applied for the solid dispersion of tranilast (*N*-(3,4-dimethoxycinnamoyl) anthranilic acid), an anti-allergic biopharmaceutics classification system (BSC) class II drug [15]. The poly[MPC-*co*-BMA] markedly improved the dissolution rate by the attractive interaction between the polymer and tranilast. Historically, polyvinylpyrrolidone (PVP) has been extensively used for solid dispersions to improve the dissolution rate without recrystallization due to the enhanced interaction in the solid state [16]. A solid dispersion containing PVP, sodium lauryl sulfate (SLS) and PTX is a good strategy for increasing the solubility and dissolution rate of PTX, with the solid dispersion formulation of PTX having been tried with the first clinical trial using oral LDM chemotherapy [11]. According to a literature review, the design of water-soluble polymers, which preferably interact with PTX to keep the amorphous states, should be considered as a crucial factor to improve the solubility and dissolution rate of PTX [17]. 

**Figure 1:**
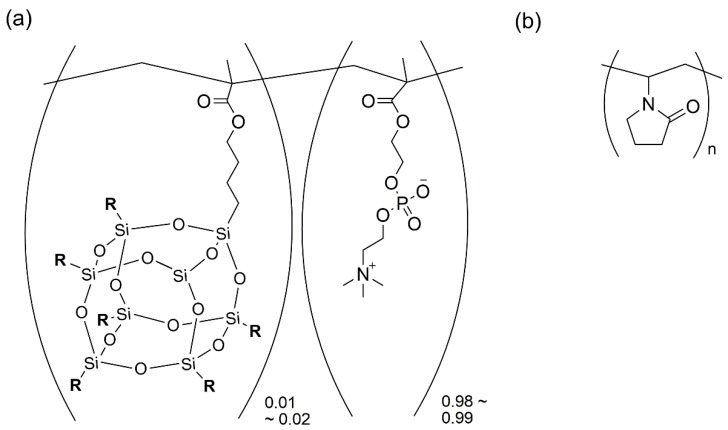
Chemical structure of (**a**) 2-methacryloyloxyethyl phosphorylcholine (MPC) and a polyhedral oligomeric silsesquioxane (POSS) methacrylate bearing an ethyl (C_2_H_5_) group (MPC-*ran*-C_2_H_5_-POSS) (R = C_2_H_5_) and (**b**) polyvinylpyrrolidone (PVP).

Based on those perspectives of the MPC-based amphiphilic copolymers, this study focused on the MPC as a hydrophilic monomer as well as the chemical structure of hydrophobic monomers, because the BMA part of poly[MPC-*co*-BMA] as mentioned above is not optimized for controlling the interaction with PTX. In a previous study, a random copolymer was synthesized by using the MPC and ethyl (C_2_H_5_) group-modified polyhedral oligomeric silsesquioxane (POSS) methacrylate (MA) as a hydrophobic monomer [18]. The obtained copolymer (MPC-*ran*-C_2_H_5_-POSS; Figure 1a) was not cytotoxic [18], and it formed a hydrophobic domain in water through the hydrophobic interaction of the C_2_H_5_-POSS moiety. In addition, it was found that the tight interaction between the C_2_H_5_-POSS and PTX was correlated with the slow release of PTX from a micelle-like assembly of MPC-*ran*-C_2_H_5_-POSS [19]. Thus, the MPC-*ran*-C_2_H_5_-POSS solubilizes PTX well, and this finding led to the idea of the application of the MPC-*ran*-C_2_H_5_-POSS for a homogeneous solid formulation. In this study, the MPC-*ran*-C_2_H_5_-POSS was investigated as a potential carrier of PTX in the formulation of a drug/polymer mixture. The solid formulation of PTX and the MPC-*ran*-C_2_H_5_-POSSS was prepared by a simple evaporation method. PVP (Figure 1b) was also added to the MPC-*ran*-C_2_H_5_-POSS as an additional hydrophilic carrier to evaluate the role of PVP in the MPC-*ran*-C_2_H_5_-POSS matrix. The solid states of PTX in the polymeric matrix were characterized by X-ray diffraction (XRD), Fourier transform infrared spectroscopy (FT-IR) and differential scanning calorimetry (DSC). The PTX dissolution behavior from the formulations was investigated and the effect of the MPC-*ran*-C_2_H_5_-POSSS on the PTX dissolution was discussed. The enhanced dissolution of PTX was found without any precipitation, which was correlated with maintaining the amorphous nature of PTX in the MPC-*ran*-C_2_H_5_-POSS/PVP matrix.

## 2. Materials and Methods 

### 2.1. Materials

The 2-methacryloyloxyethyl phosphorylcholine (MPC) was purchased from NOF Corporation (Tokyo, Japan). The MPC-*ran*-C_2_H_5_-POSS copolymer was synthesized using a previously described method (Figure S1 in Supplementary Materials) [18]. The mol. % of C_2_H_5_-POSS in the copolymer was found to be ca. 2 mol. %, which was calculated by the ^1^H NMR spectrum (Figure S2 in Supplementary Materials). PTX and 2nd Fluid for dissolution test were purchased from FUJIFILM Wako Pure Chemical Corporation, Osaka, Japan. PVP K30 (*M_n_* = 50,000) was purchased from Sigma-Aldrich Co. LLC. (St. Louis, MO, U.S.A). The other reagents and solvents were used without further purification.

### 2.2. PTX Solid Formulation Using MPC-ran-C_2_H_5_-POSS and PVP 

The formulation of MPC-*ran*-C_2_H_5_-POSS, PVP and PTX (MPC-*ran*-C_2_H_5_-POSS/PVP/PTX) was prepared using a solvent method. The MPC-*ran*-C_2_H_5_-POSS, PVP and PTX were dissolved in dehydrated ethanol (MPC-*ran*-C_2_H_5_-POSS:PVP:PTX = 44:44:12 wt. %) with continuous stirring. The solvent was removed under vacuum at room temperature. The MPC-*ran*-C_2_H_5_-POSS/PTX (C_2_H_5-_POSS-MA MPC:PTX = 88:12 wt. %) and the PTX/PVP (PVP:PTX = 88:12 wt. %) were also prepared in the same manner.

### 2.3. Dissolution Test

The dissolution of the solid formulation was tested according to the European Pharmacopoeia, using a type 2 (paddle) dissolution apparatus (NTR-6100A, Toyama Sangyo Co., Ltd., Osaka, Japan). The dissolution study was performed in 500 mL of 2nd Fluid for dissolution test mixed with 1% Tween 80 as a dissolution medium. The temperature was kept at 37 °C during the study with a stirring speed of 100 rpm. The solid dose form of 10 mg was used in this dissolution study, which contained 1.1 mg equivalent of PTX. The sample solutions (25 mL) were collected at 5, 10, 20, 30, 40, 50, 60, 90, 120 and 180 min time points and an equivalent amount of fresh media was added to maintain a constant dissolution volume. The concentration of PTX in the dissolution medium was determined by HPLC (GILSON, Middleton, WI, U.S.A.) equipped with a UV-vis detector (Gilson 119 UV/VIS Detector, GILSON, Middleton, WI, U.S.A.), two pumps (GILSON 805 Manometric Module and 306 Pump, GILSON, Middleton, WI, U.S.A.), a mixer (GILSON 811c dynamic mixer, GILSON, Middleton, WI, U.S.A.) and a column (TSKgel ODS-100S (Φ 4.6 nm × 150 mm)) from TOSOH Co., Tokyo, Japan). Before the measurements were taken, all the samples were filtered through 0.45 µm Polyvinylidene difluoride (PVDF) membrane filters. A methanol and water (70:30 v/v) mixed solution was used as the mobile phase, and a 1 mL/min flow rate was maintained during the measurements. The detection was performed at a wavelength of 227 nm. PTX release in the dissolution medium at different time intervals was calculated using the PTX standard curve. The standard solutions of concentrations 100, 50, 10, 1 and 0.1 µg/mL were prepared in methanol and the standard curve was prepared by plotting the area under the peak vs. concentration. The resulting standard curve was linear with $R^{2}=0.9949$. All the dissolution experiments were conducted in triplicate.

### 2.4. X-Ray Diffraction (XRD) 

Powder X-ray diffraction was carried out using an X-Ray diffractometer (RINT2000, Rigaku Co., Tokyo, Japan) with monochromatic CuKα radiation and a generator working at 40 kV and 20 mA. Scattering intensity was measured in the range of 2 < 2θ < 60° with scan steps of 1° min^−1^.

### 2.5. Fourier Transform Infrared Spectroscopy (FT-IR) 

The chemical bonds of the solid formulation were characterized using a Fourier transform infrared spectroscopy (FT-IR) apparatus (JASCO FT/IR-460plus, JASCO Corporation, Tokyo, Japan). The scanning wave numbers ranged from 4000 to 400 cm^−1^. KBr was used for the attenuated total reflectance crystal. The spectra were recorded from KBr pellets, prepared by mixing the formulation with KBr at room temperature. The spectrum resolution was 4 cm^−1^, and 2 mm s^−1^ scans were accumulated to determine one spectrum.

### 2.6. Differential Scanning Calorimetry (DSC) 

Thermal analyses of the formulation were carried out using a differential scanning calorimetry (DSC) apparatus (EXSTAR 6000/DSC6200, Seiko Instruments Inc., Chiba, Japan). The scan rate was 10 °C min^−1^ (first cooling, second cooling, first heating and second heating) within the temperature range of 20–185 °C. The glass transition temperature (*T_g_*) was obtained at the midpoint of change in the baseline of DSC thermograms during the second heating. The *T_g_* of the solid formulation ($T_{g\;mix}$) was calculated by using the Fox equation [11],

(1) $$1/T_{g\;mix}\;=\;\sum_{i}w_{i}/T_{g\;i}$$

where $w_{i}$ is the weight fraction of the *i*^th^ pure component and $T_{g\;i}$ is the glass transition temperature (in Kelvin) of the *i*^th^ pure component. 

## 3. Results and Discussion

### 3.1. Characterization of the PTX Solid Formulation

Three different formulations of PTX were prepared in this study at the same weight percentage of PTX in all the formulations: MPC-*ran*-C_2_H_5_-POSS:PVP:PTX = 44:44:12 wt. % (MPC-*ran*-C_2_H_5_-POSS/PVP/PTX), MPC-*ran*-C_2_H_5_-POSS:PTX = 88:12 wt. % (MPC-*ran*-C_2_H_5_-POSS/PTX) and PVP:PTX = 88:12 wt. % (PVP/PTX). In order to characterize the crystalline and amorphous states of PTX and polymers in those solid formulations, XRD, DSC and FT-IR measurements were carried out.

**Figure 2:**
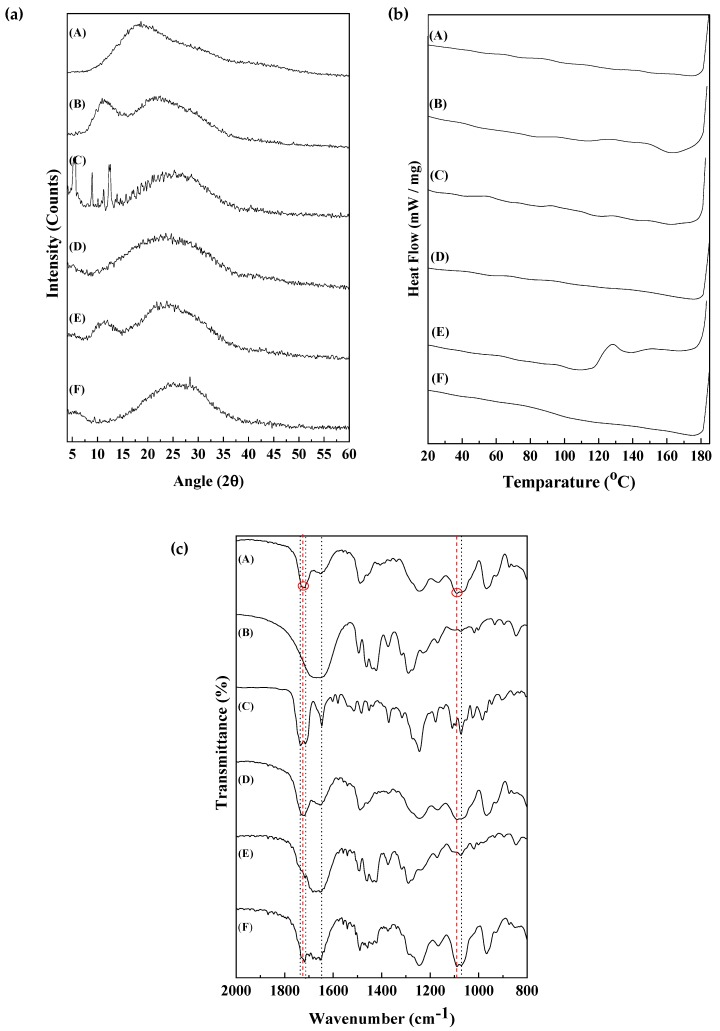
X-ray diffraction (XRD) spectra (**a**), differential scanning calorimetry (DSC) thermogram (**b**) and Fourier transform infrared spectroscopy (FT-IR) spectra (**c**) of A: MPC-*ran*-C_2_H_5_-POSS; B: PVP; C: paclitaxel (PTX); D: MPC-*ran*-C_2_H_5_-POSS/PTX; E: PVP/PTX; F: MPC-*ran*-C_2_H_5_-POSS/PVP/PTX.

XRD spectra of the MPC-*ran*-C_2_H_5_-POSS, PVP, PTX and the three different formulations are shown in Figure 2a. The semi-crystalline PTX showed diffraction peaks at 5.5°, 8.7° and 12.6° as shown in Figure 2a-C, which was consistent with the previous report [20]. The characteristic peaks of PTX as seen in Figure 2a-C were not observed in the MPC-*ran*-C_2_H_5_-POSS/PTX, PVP/PTX or the MPC-*ran*-C_2_H_5_-POSS/PVP/PTX formulations (Figure 2a-D,E,F), suggesting that the PTX existed as an amorphous state in those formulations. This finding is consistent with previous reports of PTX solid dispersion [4]. This result indicates that PTX was finely distributed over the MPC-*ran*-C_2_H_5_-POSS/PVP/PTX in the solid homogeneous matrix [11,21].

From the DSC thermograms (Figure 2b and Figure S3 in the Supplementary Materials), *T_g_* of the solid formulations and the constituent polymers were calculated, and the results supported the results of the XRD. The *T_g_* of MPC-*ran*-C_2_H_5_-POSS, PVP and PTX were observed at 70 °C (343 K), 156 °C (429 K) and 106 °C (379 K), respectively. The detailed calculation of *T_g_* is shown in Figure S3-A–C in the Supplementary Materials. Since the obtained *T_g_* of PVP was consistent with the findings of a previous study [22], the calculation of *T_g_* values from the second heating curves was reliable. It is known that PTX exhibits a melting point at 213–217 °C as a semi-crystal [23]. However, it also bears a *T_g_* in an amorphous state [24]. The MPC-*ran*-C_2_H_5_-POSS/PVP/PTX solid formulation showed a single *T_g_* at 91 °C (364 K) (Figure 2b-F and Figure S3-F in Supplementary Materials). 

According to the Fox equation (Equation (1)), *T_g_* *_mix_* of the MPC-*ran*-C_2_H_5_-POSS/PVP/PTX was calculated to be ca. 98 °C (381 K). Equation (1) only allows the prediction from the properties of pure components; the asymmetric entropic and enthalpic contribution are not reflected in this equation. In addition, the Equation (1) does not reflect the strength of intercomponent and intracomponent interaction, the composition-dependent energetic contribution from hetero-contact, the entropic effect, or the structural heterogeneity term. Taking the limitation of the Equation (1) into account, it is difficult to directly compare *T_g_* with *T_g_* *_mix_*.

In the case of amorphous binary systems, the microstructure is closely related to the glass transition temperature, and the microstructural characteristics become evident in miscibility studies of binary polymer and drug/polymer mixtures (in this case, the MPC-*ran*-C_2_H_5_-POSS/PVP/PTX system) [25,26]. Brostow et al. proposed an analytical equation (the Brostow, Chiu, Kalogeras and Vassilikou-Dova (BCKV) equation) for predicting *T_g_* that characterizes the binary systems as polymer blends or copolymers depending on their composition [26]:

(2) $$T_{g}=\Delta T_{g}+x_{1}T_{g1}+\left(1-x_{1}\right)T_{g2}$$

(3) $$\Delta T_{g}=x_{1}\left(1-x_{1}\right)\left[a_{0}+a_{1}\left(2x_{1}-1\right)+a_{2}{\left(2x_{1}-1\right)}^{2}\right]$$

where $\Delta T_{g}$ is the deviation from the linear function (the Fox equation), *x_i_* is the mass fraction of component *i* and $a_{i}$ represents the parameters of a polynomial (a quadratic polynomial for binary systems). In the proposed Equations (2) and (3), the parameters of $a_{i}$ should be determined to correlate the experimental data, Moreover, they highlighted the complexity of such binary systems [26]. The high deviation between the experimental *T_g_* values and the predicted values calculated by the Equation (1) is evidence of the increased complexity; the parameters of $a_{i}$ in the equation reflect the differences between the strength of intercomponent and intracomponent interactions, composition-dependent energetic contributions from hetero-contacts, entropic effects and structural heterogeneities [25]. The BCKV equation was also used to correlate the experimental data from the investigation on the drug/polymer mixture, poly(vinyl pyrrolidone-*co*-vinyl acetate), and the results agreed well with the experimental data [27]. 

According to the abovementioned perspectives of the complicated *T_g_* estimation, the BCKV equation was applied to evaluate the formulation of the MPC-*ran*-C_2_H_5_-POSS/PVP/PTX. Table 1 shows the *T_g_* values; the *T_gDSC_* was determined by the DSC measurements (see Figure S3), and the *T_g mix_* was calculated by the Fox equation. The $\Delta T_{g}$ values, which were the difference between the *T_gDSC_* and *T_g mix_* of the MPC-*ran*-C_2_H_5_-POSS/PTX, the PVP/PTX and the MPC-*ran*-C_2_H_5_-POSS/PVP/PTX, were −14, −45 and −17 K, respectively. According to Equation (3), the $\Delta T_{g}$ values included the parameters of *a_0_*–*a*_2_ (in the case of MPC-*ran*-C_2_H_5_-POSS/PVP/PTX, *a_0_*–*a*_3_ due to the trinary systems). Since the parameters of *a_i_* reflected the differences of the interaction energies and the structural heterogeneities as mentioned previously, those solid formulations exhibited some energetic matters of the interactions between the MPC-*ran*-C_2_H_5_-POSS, PVP and PTX. However, the smaller $\Delta T_{g}$ values of the MPC-*ran*-C_2_H_5_-POSS/PTX and the MPC-*ran*-C_2_H_5_-POSS/PVP/PTX compared to those of the PVP/PTX suggest that the MPC-*ran*-C_2_H_5_-POSS contributed to the increased miscibility of PTX. 

Since the *T_g_* of the binary polymer and drug/polymer mixture is dependent on the mass fraction, the observed single *T_g_* at 91 °C, which was higher than the value for the MPC-*ran*-C_2_H_5_-POSS (70 °C), follows conventional miscible organic blends [25]. In other words, the obtained single *T_g_* value suggests the miscibility and strong interaction between PTX, PVP and polymers in the single homogeneous phase [11]. In addition, the increase of the *T_g_* of the MPC-*ran*-C_2_H_5_-POSS (70 °C) to 91 °C suggests the increased stability of PTX by the strong interaction in the MPC-*ran*-C_2_H_5_-POSS/PVP/PTX homogeneous formulation [24]. 

From the FT-IR spectrum of PTX (Figure 2c-C), strong carbonyl bands were observed at 1715 cm^−1^ (C=O, ketone) and 1734 cm^−1^ ((C=O)-O-, ester) in addition to 1644 cm^−1^ (aromatics) and 1070 cm^−1^ (C-O-C). For the MPC-*ran*-C_2_H_5_-POSS, the corresponding ester bonds resulted in the appearance of the peaks at 1724 cm^−1^ ((C=O)-O-, ester) and 1080 cm^−1^ (P-O) in the MPC part [28] (Figure 2c-A). In the case of the MPC-*ran*-C_2_H_5_-POSS/PTX (Figure 2c-D), the similar spectrum of the MPC-*ran*-C_2_H_5_-POSS was observed. This suggested that the incorporated PTX in the MPC-*ran*-C_2_H_5_-POSS matrix was difficult to confirm from the IR spectrum; the FT-IR spectrum of the MPC-*ran*-C_2_H_5_-POSS/PTX was governed by the MPC-*ran*-C_2_H_5_-POSS. Besides, when PVP was added to the matrix (MPC-*ran*-C_2_H_5_-POSS/PVP/PTX), many jagged peaks around 1705–1740 cm^−1^ were observed (Figure 2c-F). This suggests the restricted molecular interaction between individual components, which was consistent with the DSC data indicating a high $T_{g\;mix}$ of the solid formulation. This result also indicated that the PVP contributed to the reduction of crystallinity in the formulation. The FT-IR measurements of the physical mixture of the MPC-*ran*-C_2_H_5_-POSS/PTX, PVP/PTX and MPC-*ran*-C_2_H_5_-POSS/PVP/PTX were performed to confirm the detection of PTX in the FT-IR spectra; carbonyl bands at 1715 cm^−1^ (C=O, ketone) and 1734 cm^−1^ ((C=O)-O-, ester) in addition to 1644 cm^−1^ (aromatics) and 1070 cm^−1^ (C-O-C) at 12 wt. % (the same weight ratio; Figure S4 in the Supplementary Materials). The results of the FT-IR spectra of the physical mixture showed that the spectra were a summation of the individual components. The results suggest the negligible interactions between the components in combination. Thus, in the formulation, PTX was more homogeneously distributed over the MPC-*ran*-C_2_H_5_-POSS/PVP matrix than in the physical mixture [11]. Taking all the information of XRD, DSC and FT-IR spectra into account, PTX existed as an amorphous state in the single phase homogeneous formulation.

### 3.2. Dissolution Behavior of PTX from the Formulation

**Figure 3:**
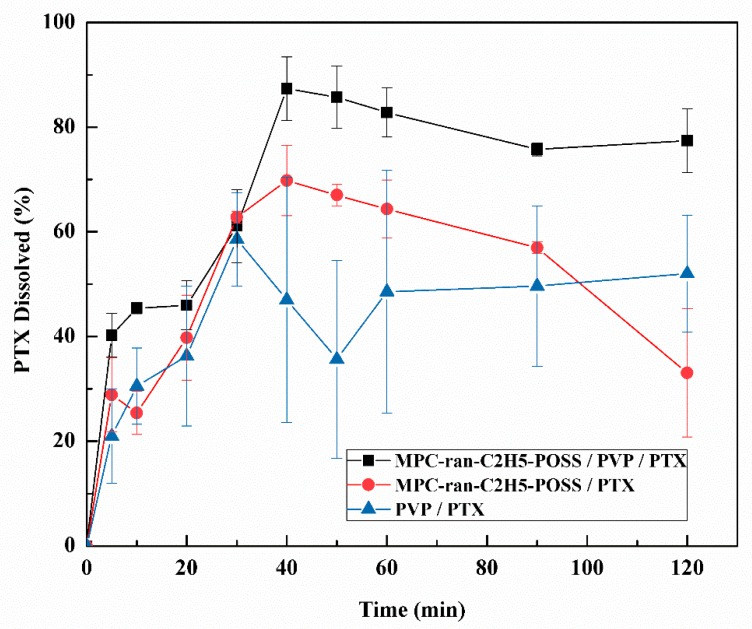
Dissolution profiles of PTX formulations in 2nd Fluid for dissolution test mixed with 1% Tween 80 at 37 °C; (■): MPC-*ran*-C_2_H_5_-POSS/PVP/PTX; (●): MPC-*ran*-C_2_H_5_-POSS/PTX; and (▲)): PVP/PTX of solid formulations. Values are means ± S.D. (n = 3)**.**

Dissolution tests were carried out in order to assess the dissolution of PTX from the MPC-*ran*-C_2_H_5_-POSS/PVP/PTX, MPC-*ran*-C_2_H_5_-POSS/PTX and PVP/PTX. As shown in Figure 3, the dissolution rate of PTX from the MPC-*ran*-C_2_H_5_-POSS/PVP/PTX was much faster than that from the MPC-*ran*-C_2_H_5_-POSS/PTX and PVP/PTX for the first 10 min. Here, the dissolution rate was calculated from the slope of the first line curve between 0 to 10 min. The cumulative amount of PTX from the MPC-*ran*-C_2_H_5_-POSS/PVP/PTX was saturated until 20 min, and then the dissolution was accelerated. As a result, PTX was around 90% dissolved at 40 min. Since the complicated dissolution behavior was not observed in the case of the MPC-*ran*-C_2_H_5_-POSS/PTX and the PVP/PTX, the first stage of the dissolution up until 20 min was due to the synergistic effects of the MPC-*ran*-C_2_H_5_-POSS and PVP as the matrix. The second stage from 20 min to 40 min was governed by the MPC-*ran*-C_2_H_5_-POSS because the apparent dissolution behavior was similar to the MPC-*ran*-C_2_H_5_-POSS/PTX. The maximum PTX dissolution from the MPC-*ran*-C_2_H_5_-POSS/PVP/PTX was ~2 µg mL^−1^ (an almost complete release of PTX) over 40 min. A similar phenomenon was observed in the case of the MPC-*ran*-C_2_H_5_-POSS/PTX, where the solubility of PTX was ~1.5 µg/mL. It is noted that the dissolved concentration percentage slowly decreased after reaching the maximum percentage. The general phenomenon of the homogeneous solid formulation was as follows—the decrease in the PTX concentration after a certain time was due to the fact that the crystal nucleation rate of PTX rises according to the ratio of the supersaturated solubility to the solubility of the crystal [29]. 

The increase in the apparent dissolution from the MPC-*ran*-C_2_H_5_-POSS/PVP/PTX was caused by several factors such as the amorphous state of PTX in the formulations, Figure 2a-D,F, an enhanced solubilizing effect of the MPC-*ran*-C_2_H_5_-POSS (PTX solubility in the MPC-*ran*-C_2_H_5_-POSS 100 mg mL^-1^ solution was found to be 75.5 ± 8.8 µg) [19], the inhibited recrystallization of amorphous PTX by PVP and so on. Since the MPC-*ran*-C_2_H_5_-POSS exhibited an amphiphilic nature [19], the presence of the MPC-*ran*-C_2_H_5_-POSS could prevent the immediate recrystallization of amorphous PTX at the water interface to reduce interfacial tension between PTX and water [30]. In the case of the PVP/PTX, the amount of PTX dissolution was found to be lower than that in the MPC-*ran*-C_2_H_5_-POSS/PVP/PTX and the MPC-*ran*-C_2_H_5_-POSS/PTX, suggesting that the absence of hydrophobic moieties in the hydrophilic PVP matrix somewhat induced the recrystallization of PTX. Thus, the incorporation of the MPC-*ran*-C_2_H_5_-POSS in the solid formulation of PTX plays an important role in modulating the dissolution behavior of PTX, which leads to PTX supersaturation throughout the media [31].

## 4. Conclusions

MPC-*ran*-C_2_H_5_-POSS was incorporated into a solid formulation of PTX with PVP. The MPC-*ran*-C_2_H_5_-POSS/PVP/PTX single-phase homogeneous formulation was found to enhance the dissolution rate of PTX. The improved wetting of the formulation accompanied by the amphiphilic nature of the MPC-*ran*-C_2_H_5_-POSS facilitated rapid and complete drug release (approximately 90% within 40 min). The increased interaction between PTX and the polymeric matrix of the MPC-*ran*-C_2_H_5_-POSS/PVP/PTX formulation is likely to reduce the mobility of amorphous PTX, which results in the enhanced dissolution of PTX. Therefore, the formulation of PTX in combination with MPC-*ran*-C_2_H_5_-POSS and PVP would be a promising approach to enhance the physicochemical properties of PTX. The results obtained encourage further investigation of the pharmacokinetic parameters in an animal model.

## Figures and Tables

**Table 1:** The glass transition temperature *T_gDSC_*, the *T_g mix_* and the $\Delta T_{g}$ values of the formulations, the constituent polymers and PTX.

Sample Codes	$T_{gDSC}{}^{a}$ (K)	$T_{gmix}{}^{b}$ (K)	$\Delta T_{g}$ $(=\;T_{gDSC\;}-$ $T_{gmix})\;(K)$
MPC-*ran*-C_2_H_5_-POSS	343	-	-
PVP	429	-	-
PTX	379	-	-
MPC-*ran*-C_2_H_5_-POSS/PTX	333	347	−14
PVP/PTX	377	422	−45
MPC-*ran*-C_2_H_5_-POSS/PVP/PTX	364	381	−17

^a^ Experimentally determined *T_g_* values from DSC data. ^b^ Calculated by Equation (1).

## Supplementary Materials

The following are available online at https://www.mdpi.com/1996-1944/12/7/1058/s1, Figure S1: Synthesis of MPC-*ran*-C_2_H_5_-POSS, Figure S2: A ^1^H NMR spectrum of MPC-*ran*-C_2_H_5_-POSS in methanol-*d*_4_., Figure S3: DSC thermograms of A: MPC-*ran*-C_2_H_5_-POSS; B: PVP; C: PTX; D: MPC-*ran*-C_2_H_5_-POSS/PTX; E: PVP/PTX; F: MPC-*ran*-C_2_H_5_-POSS/PVP/PTX, Figure S4: FT-IR spectra of A: PTX; B: MPC-*ran*-C_2_H_5_-POSS/PTX physical mixture; C: PVP/PTX physical mixture; D: MPC-*ran*-C_2_H_5_-POSS/PTX /PVP/PTX physical mixture.

## References

[1] Gelderblom H., Verweij J., Nooter K., Sparreboom A. (2001). Cremophor EL: The drawbacks and advantages of vehicle selection for drug formulation. Eur. J. Cancer.

[2] Alam M.A., Ali R., Al-Jenoobi F.I., Al-Mohizea A.M. (2012). Solid dispersions: A strategy for poorly aqueous soluble drugs and technology updates. Expert Opin. Drug Deliv..

[3] Bikiaris D.N. (2011). Solid dispersions, Part I: Recent evolutions and future opportunities in manufacturing methods for dissolution rate enhancement of poorly water-soluble drugs. Expert Opin. Drug Deliv..

[4] Shen Y.Q., Lu F., Hou J.W., Shen Y.Y., Guo S.R. (2013). Incorporation of paclitaxel solid dispersions with poloxamer188 or polyethylene glycol to tune drug release from poly(epsilon-caprolactone) films. Drug Dev. Ind. Pharm..

[5] Yang T., Cui F.D., Choi M.K., Lin H.X., Chung S.J., Shim C.K., Kim D.D. (2007). Liposome formulation of paclitaxel with enhanced solubility and stability. Drug Deliv..

[6] Hu X.S., Lin L., Xing P.Y., Zhang C.G., Wang L., Liz Y.Q. (2017). The Clinical Efficacy and Safety of Paclitaxel Liposome on the Patients with Non-Small Cell Lung Cancer: A Meta-Analysis. J. Thorac. Oncol..

[7] Bernabeu E., Cagel M., Lagomarsino E., Moretton M., Chiappetta D.A. (2017). Paclitaxel: What has been done and the challenges remain ahead. Int. J. Pharm..

[8] Louage B., De Wever O., Hennink W.E., De Geest B.G. (2017). Developments and future clinical outlook of taxane nanomedicines. J. Control. Release.

[9] Bouquet W., Ceelen W., Fritzinger B., Pattyn P., Peeters M., Remon J.P., Vervaet C. (2007). Paclitaxel/beta-cyclodextrin complexes for hyperthermic peritoneal perfusion—Formulation and stability. Eur. J. Pharm. Biopharm..

[10] Hamada H., Ishihara K., Masuoka N., Mikuni K., Nakajima N. (2006). Enhancement of water-solubility and bioactivity of paclitaxel using modified cyclodextrins. J. Biosci. Bioeng..

[11] Moes J., Koolen S., Huitema A., Schellens J., Beijnen J., Nuijen B. (2013). Development of an oral solid dispersion formulation for use in low-dose metronomic chemotherapy of paclitaxel. Eur. J. Pharm. Biopharm..

[12] Yang F.H., Zhang Q., Liang Q.Y., Wang S.Q., Zhao B.X., Wang Y.T., Cai Y., Li G.F. (2015). Bioavailability Enhancement of Paclitaxel via a Novel Oral Drug Delivery System: Paclitaxel-Loaded Glycyrrhizic Acid Micelles. Molecules.

[13] Yusa S.I., Fukuda K., Yamamoto T., Ishihara K., Morishima Y. (2005). Synthesis of well-defined amphiphilic block copolymers having phospholipid polymer sequences as a novel blocompatible polymer micelle reagent. Biomacromolecules.

[14] Konno T., Watanabe J., Ishihara K. (2003). Enhanced solubility of paclitaxel using water-soluble and biocompatible 2-methacryloyloxyethyl phosphorylcholine polymers. J. Biomed. Mater. Res. A.

[15] Onoue S., Kojo Y., Suzuki H., Yuminoki K., Kou K., Kawabata Y., Yamauchi Y., Hashimoto N., Yamada S. (2013). Development of novel solid dispersion of tranilast using amphiphilic block copolymer for improved oral bioavailability. Int. J. Pharm..

[16] Taylor L.S., Zografi G. (1997). Spectroscopic characterization of interactions between PVP and indomethacin in amorphous molecular dispersions. Pharm. Res..

[17] Vo C.L.N., Park C., Lee B.J. (2013). Current trends and future perspectives of solid dispersions containing poorly water-soluble drugs. Eur. J. Pharm. Biopharm..

[18] Chatterjee S., Matsumoto T., Nishino T., Ooya T. (2018). Tuned Surface and Mechanical Properties of Polymeric Film Prepared by Random Copolymers Consisting of Methacrylate-POSS and 2-(Methacryloyloxy)ethyl Phosphorylcholine. Macromol. Chem. Phys..

[19] Chatterjee S., Ooya T. (2018). Hydrophobic Nature of Methacrylate-POSS in Combination with 2-(Methacryloyloxy)ethyl Phosphorylcholine for Enhanced Solubility and Controlled Release of Paclitaxel. Langmuir.

[20] Liu X., Lei L., Hou J.W., Tang M.F., Guo S.R., Wang Z.M., Chen K.M. (2011). Evaluation of two polymeric blends (EVA/PLA and EVA/PEG) as coating film materials for paclitaxel-eluting stent application. J. Mater. Sci. Mater. Med..

[21] Han H.K., Lee B.J., Lee H.K. (2011). Enhanced dissolution and bioavailability of biochanin A via the preparation of solid dispersion: In vitro and in vivo evaluation. Int. J. Pharm..

[22] Chan S.Y., Chung Y.Y., Cheah X.Z., Tan E.Y.L., Quah J. (2015). The characterization and dissolution performances of spray dried solid dispersion of ketoprofen in hydrophilic carriers. Asian J. Pharm. Sci..

[23] Lee J., Choi J.Y., Park C.H. (2008). Characteristics of polymers enabling nano-comminution of water-insoluble drugs. Int. J. Pharm..

[24] Richard R.E., Schwarz M., Ranade S., Chan A.K., Matyjaszewski K., Sumerlin B. (2005). Evaluation of acrylate-based block copolymers prepared by atom transfer radical polymerization as matrices for paclitaxel delivery from coronary stents. Biomacromolecules.

[25] Kalogeras I.M., Haag Lobland H.E. (2012). The Nature of the Glassy State: Structure and Glass Transitions. J. Mater. Educ..

[26] Brostow W., Chiu R., Kalogeras I.M., Vassilikou-Dova A. (2008). Prediction of glass transition temperatures: Binary blends and copolymers. Mater. Lett..

[27] Babu R.J., Brostow W., Fasina O., Kalogeras I.M., Sathigari S., Vassilikou-Dova A. (2011). Encapsulation of Hydrophobic Drugs in a Copolymer: Glass Transition Behavior and Miscibility Evaluation. Polym. Eng. Sci..

[28] Tateishi T., Kyomoto M., Kakinoki S., Yamaoka T., Ishihara K. (2014). Reduced platelets and bacteria adhesion on poly(ether ether ketone) by photoinduced and self-initiated graft polymerization of 2-methacryloyloxyethyl phosphorylcholine. J. Biomed. Mater. Res. A.

[29] Que C.L., Gao Y., Raina S.A., Zhang G.G.Z., Taylor L.S. (2018). Paclitaxel Crystal Seeds with Different Intrinsic Properties and Their Impact on Dissolution of Paclitaxel-HPMCAS Amorphous Solid Dispersions. Cryst. Growth Des..

[30] Aggarwal A.K., Singh S. (2011). Physicochemical characterization and dissolution study of solid dispersions of diacerein with polyethylene glycol 6000. Drug Dev. Ind. Pharm..

[31] Miller J.M., Beig A., Carr R.A., Spence J.K., Dahan A. (2012). A Win-Win Solution in Oral Delivery of Lipophilic Drugs: Supersaturation via Amorphous Solid Dispersions Increases Apparent Solubility without Sacrifice of Intestinal Membrane Permeability. Mol. Pharm..

