# Supplementary material for: Amphiphilic Copolymer of Polyhedral Oligomeric Silsesquioxane (POSS) Methacrylate for Solid Dispersion of Paclitaxel
Source: Materials (Basel). 2019 Mar 30;12(7):1058. doi: 10.3390/ma12071058 (PMC6479374; doi:10.3390/ma12071058)
Supplement: Supplementary file 1 [file materials-12-01058-s001.pdf]

materials

Supplementary Materials

Amphiphilic Copolymer of Polyhedral Oligomeric Silsesquioxane (POSS) Methacrylate for Solid Dispersion of Paclitaxel

Suchismita Chatterjee and Tooru Ooya *

Graduate School of Engineering, Department of Chemical Science and Engineering, Kobe University; Kobe, 657-8501, Japan, suchismita@stu.kobe-u.ac.jp

* Correspondence: ooya@tiger.kobe-u.ac.jp; Tel.: +81-78-803-6255

Received: 21 February 2019; Accepted: 27 March 2019; Published: date

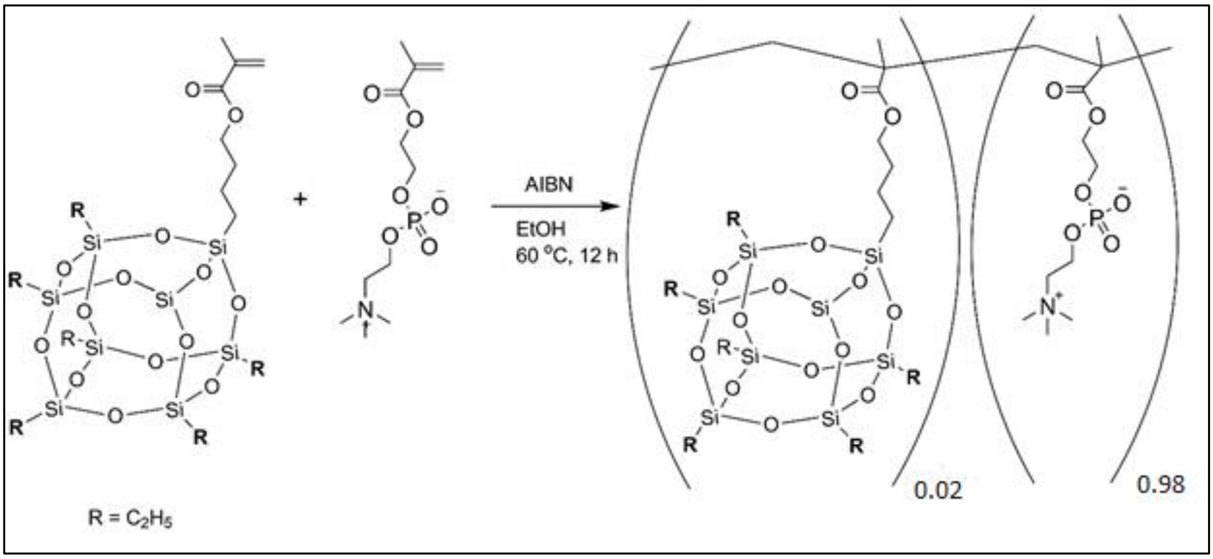

Figure S1. Synthesis of MPC-*ran*-C_2H_5-POSS.

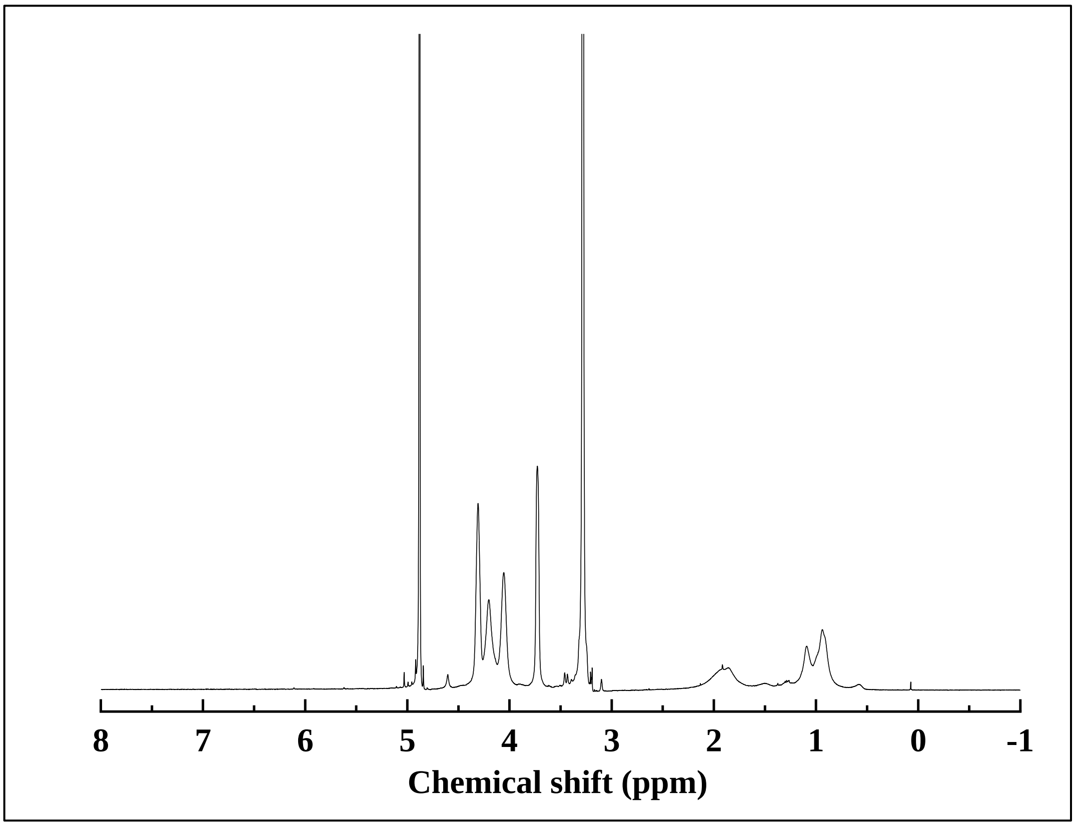

Figure S2. A ^{1}H NMR spectrum of MPC-*ran*-C_2H_5-POSS in methanol-*d*4. .

^{1}H NMR (400 MHz) spectra obtained from methanol-*d*4 δ: 0.94 [s, -C-CH_3], 1.91 [s, -CH_2-], 3.28 [s, -N$(CH_3)_3$], 3.9-4.3 [m, -OCH_2], 3.76 [s, -CH_2N], 0.59 [s, -Si-CH_2-].

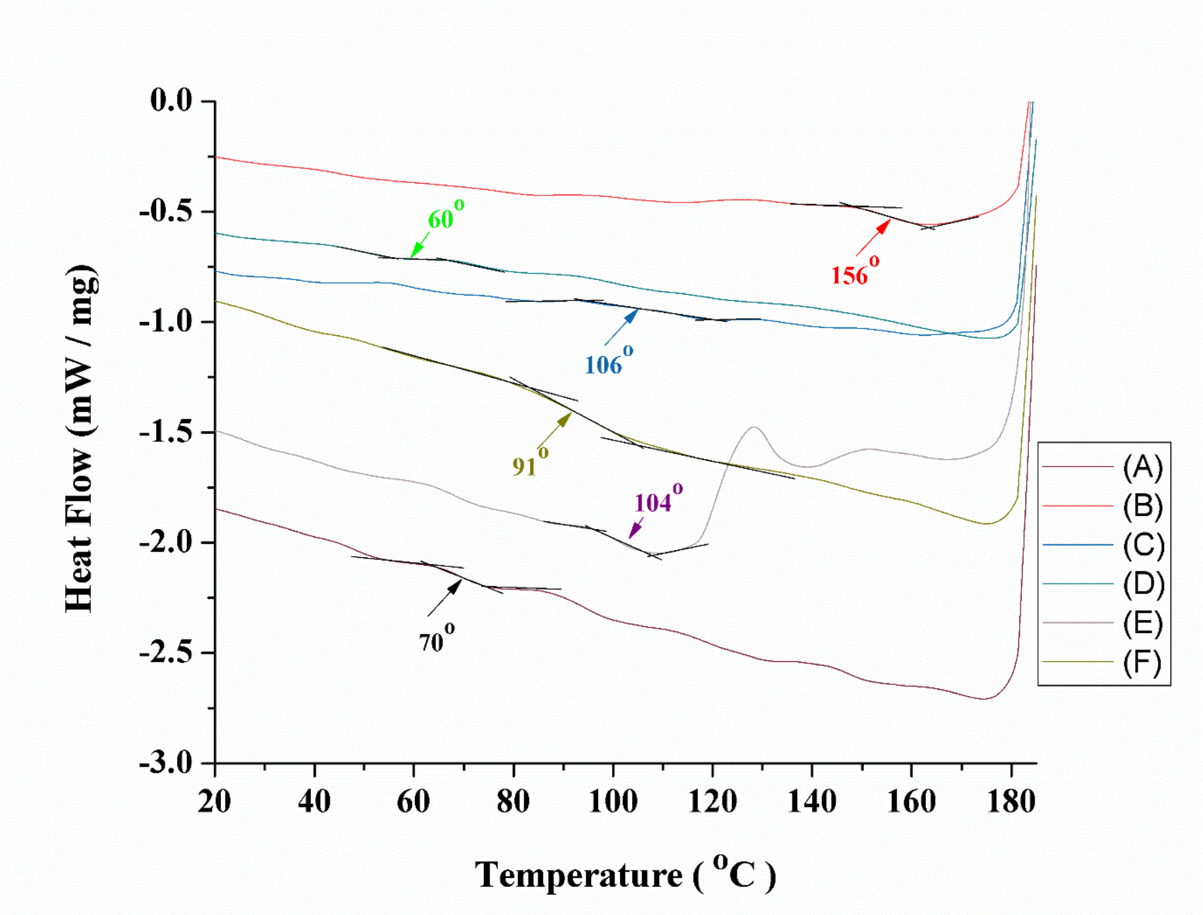

Figure S3. DSC thermograms (the 2nd heating) of A: MPC-*ran*-C_2H_5-POSS; B: PVP; C: PTX; D: MPC-*ran*-C_2H_5-POSS/PTX; E: PVP/PTX; F: MPC-*ran*-C_2H_5-POSS/PVP/PTX.

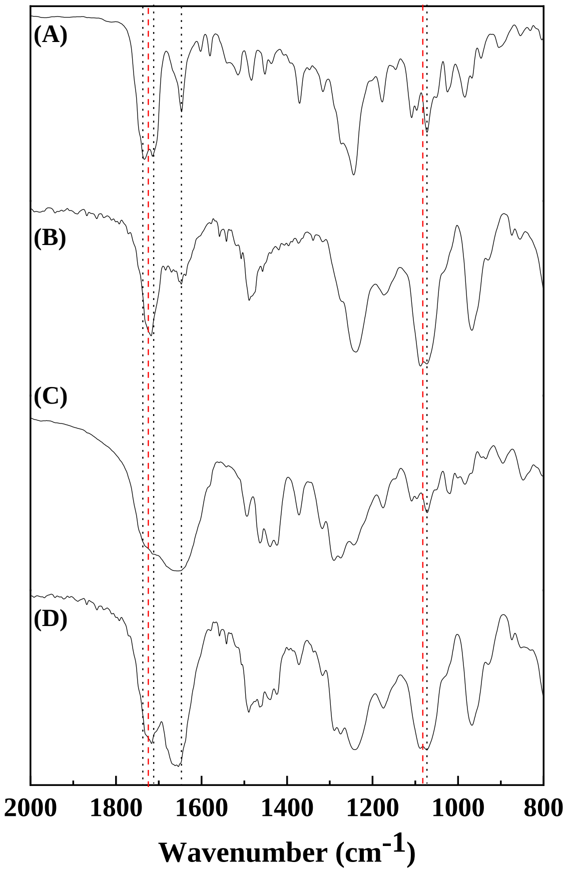

Figure S4. FT-IR spectra of A: PTX; B: MPC-*ran*-C_2H_5-POSS/PTX physical mixture; C: PVP/PTX physical mixture; D: MPC-*ran*-C_2H_5-POSS/PTX /PVP/PTX physical mixture.
